# Support vector machines classifiers of physical activities in preschoolers

**DOI:** 10.1002/phy2.6

**Published:** 2013-06-07

**Authors:** Wei Zhao, Anne L Adolph, Maurice R Puyau, Firoz A Vohra, Nancy F Butte, Issa F Zakeri

**Affiliations:** 1 Department of Epidemiology and Biostatistics, Drexel UniversityPhiladelphia, Pennsylvania, 19120; 2 USDA/ARS Children's Nutrition Research Center, Department of Pediatrics, Baylor College of MedicineHouston, Texas, 77030

**Keywords:** Accelerometers, activity monitoring, classification, multinomial logistic regression classifiers, support vector machines classifiers

## Abstract

The goal of this study is to develop, test, and compare multinomial logistic regression (MLR) and support vector machines (SVM) in classifying preschool-aged children physical activity data acquired from an accelerometer. In this study, 69 children aged 3–5 years old were asked to participate in a supervised protocol of physical activities while wearing a triaxial accelerometer. Accelerometer counts, steps, and position were obtained from the device. We applied *K*-means clustering to determine the number of natural groupings presented by the data. We used MLR and SVM to classify the six activity types. Using direct observation as the criterion method, the 10-fold cross-validation (CV) error rate was used to compare MLR and SVM classifiers, with and without sleep. Altogether, 58 classification models based on combinations of the accelerometer output variables were developed. In general, the SVM classifiers have a smaller 10-fold CV error rate than their MLR counterparts. Including sleep, a SVM classifier provided the best performance with a 10-fold CV error rate of 24.70%. Without sleep, a SVM classifier-based triaxial accelerometer counts, vector magnitude, steps, position, and 1- and 2-min lag and lead values achieved a 10-fold CV error rate of 20.16% and an overall classification error rate of 15.56%. SVM supersedes the classical classifier MLR in categorizing physical activities in preschool-aged children. Using accelerometer data, SVM can be used to correctly classify physical activities typical of preschool-aged children with an acceptable classification error rate.

## Introduction

Novel approaches to classify physical activities in young children are essential for identifying their characteristically sporadic physical activity patterns. Because of methodological limitations, there is a paucity of quantitative data on the habitual physical activity patterns in preschool-aged children. Cost-effective, nonintrusive, valid, and precise methods for the classification of physical activities in preschool-aged children are essential to determine physical activity behaviors, prevalence and determinants, dose–response relationships between physical activity and health outcomes, and intervention effectiveness. Accelerometers are used for activity recognition using body-mounted sensors; however, the mathematical modeling of accelerometer counts in preschool-aged children has been limited to regression models that do not take into account the interdependence of the data and do not exploit all the information.

Statistically sophisticated models have extracted more information from the accelerometer signal in studies in adults and school-aged children. Neural networks (Kiani et al. 1998; Rothney et al. 2007; Staudenmayer et al. 2009), multivariate adaptive regression splines (Zakeri et al. 2010), cross-sectional time series (Zakeri et al. 2008), and decision trees (Brage et al. 2004; Tapia et al. 2007; Bonomi et al. 2009) have been used to estimate energy expenditure from accelerometers. Others have used pattern recognition techniques for classification of physical activities (Companjen 2009). Quadratic discriminant analysis (QDA) and hidden Markov model (HMM) were trained to recognize four activities (walking, walking uphill, vacuuming, and working on a computer) in six adults (Pober et al. 2006). The recognition accuracy was 55% for walking, 58% for walking uphill, 68% for vacuuming, and 100% for working on a computer. Support vector machines (SVM) models of triaxial accelerometry and photography were used to classify nine common lifestyle activities in adults and achieved 93% accuracy (Cho et al. 2008). SVM was also applied to running, standing, jumping, and walking in 11 adults, with a recognition accuracy of 92% (He and Jin 2008). Artificial neural network (ANN) based on uniaxial accelerometer worn on the hip or ankle in 49 adults achieved an accuracy of 80.4% and 77.7% for hip and ankle placement, respectively; misclassification was highest with stair climbing (25–60%) and standing still (6–26%) (De Vries et al. 2011b).

ANN models have been used to identify types of physical activity (sitting, standing, walking, running, rope skipping, playing, soccer, and cycling) in school-aged children using uniaxial and triaxial accelerometry on the hip and ankle (De Vries et al. 2011a). ANN models using the hip placement accurately predicted activities 72.4% and 76.8% of the time using uniaxial and triaxial accelerometers, respectively. The recognition accuracy was lower with the ankle placement (57.3% and 68.2%). Most misclassification occurred with standing, sitting, and cycling.

In this study, we use triaxial accelerometers and apply sophisticated mathematical modeling techniques, multinomial logistic regression (MLR) and SVM, for the first time to classify physical activities in preschool-aged children. MLR and SVM models are developed in 69 preschool-aged children using direct observation as the criterion method. Applying advanced modeling techniques will result in improved population-specific models for the classification of physical activities from triaxial accelerometry that can be easily implemented using standard software packages.

The specific aims of this study are to develop, test, and compare algorithms using MLR and SVM methods based on triaxial accelerometry for the classification of physical activities in preschool-aged children.

## Material and Methods

### Study design

A cross-sectional study design was used in which preschool-aged children participated in a protocol of planned physical activities under constant observation. The protocol entailed a 7-h visit to the Children's Nutrition Research Center metabolic research unit. While inside a room respiration calorimeter, the child was instructed to follow a protocol of physical activities designed to characterize the range of energy expenditure and physical movement typical of this age group. Using direct observation as the criterion method, MLR and SVM models for the classification of physical activities based on triaxial accelerometry were developed, tested, and compared in preschool-aged children.

The Institutional Review Board for Human Subject Research for Baylor College of Medicine and Affiliated Hospitals approved the protocol. All parents gave written informed consent to participate in this study.

### Subjects

A total of 69 preschool-aged children, balanced for age and gender, were enrolled. All participants were healthy, 3- to 5-year-old children. Twenty percent of the children were classified as overweight or obese, according to the Centers for Disease Control and Prevention (Kuczmarski et al. 2000). Children on prescription drugs or with chronic diseases including metabolic or endocrine disorders, asthma treated with steroids, sleep apnea, and any condition that interfered with physical activity were excluded from the study. Informed consent was obtained from all parents/primary caretakers prior to enrollment in the study.

### Accelerometry

ActiGraph GT3X+ activity monitor (ActiGraph, Pensacola, FL), a triaxial accelerometer, was used to measure the amount and frequency of movement of the children. GT3X+ monitors were affixed above the iliac crest of the right hip with an adjustable elastic belt. GT3X+ monitor is compact and lightweight, measuring 4.6 cm × 3.3 cm × 1.5 cm with a weight of 19 g. The GT3X+ output includes activity counts on the vertical (act_X), horizontal (act_Y), and diagonal (act_Z) axes, vector magnitude which is equal to sqrt(act_X^2^ + act_Y^2^ +act_Z^2^), and number of steps taken. The GT3X+ has an inclinometer to determine subject position (0 = monitor off or person lying on his/her side; 1 = standing; 2 = lying down; 3 = sitting) and to identify periods when the device has been removed. GT3X+ records time varying accelerations ranging in magnitude from ±6 g's. The accelerometer output is sampled by a 12-bit analog to digital convertor, set at 30 Hz for our application. The digital filter band limits the accelerometer to the frequency range from 0.25 to 2.5 Hz, which has been carefully chosen to detect normal human motion and to reject changing accelerations outside the pass band. Each sample was summed over a 60-sec epoch.

### Protocol

All children were asked to perform a series of physical activities while in the calorimeter in the same order between 9:00 am and 4:00 pm under staff supervision. In between the series of scheduled physical activities, the children were given “free-time” to engage in light activities of their choice. The staff recorded minute-to-minute observations of the child's activities. The children were given lunch at 11:30 am outside the calorimeter, and snacks at 9:30 am and 2:30 pm inside the calorimeter. The protocol included the following discrete physical activities:

Sleep: Children slept on a children's bed for 45–120 min after lunch.
Watching TV: Children reclined against a pillow and watched a movie on TV for 20 min.
Coloring: Children sat in a chair at a desk drawing with crayons for 10 min.
Video games: Children while sitting played video games for 10 min.
Puzzles: Children while sitting on the floor assembled puzzles for 10 min.
Kitchen/toys: Children while standing played at a child's kitchen or with other toys (trucks, blocks, etc.) for 15 min.
Ball toss: Children while standing repeatedly threw balls at targets across the room and walked quickly to retrieve them for 15 min.
Active video game: Children while standing on a video game mat played a variety of motion games for 10 min.
Dance: While following an instructor in a video displayed on a television screen, children performed a variety of dances for 15 min.
Aerobics: While following an instructor in a video displayed on a television screen, children performed a variety of aerobic activities for 15 min.
Running in place: Children ran in place on a game mat while competing in a video race displayed on a television screen for 6 min.

### Statistical methods

In order to determine the number of the natural groupings of physical activities presented by the minute-to-minute data, *K*-means clustering (with Euclidean distance as the distance function) was first conducted. The result of the *K*-means clustering was used as evidence to recategorize the data. We applied MLR and SVM classifiers to the data. The main input features used in the classifiers were activity counts, vector magnitude, the number of steps taken, position, and their 1-min and 2-min lag and lead values. Since the sleep period can be accurately removed from the data, we also applied SVM classification models to the data without the sleep period. We compared the 10-fold CV error rates of the classifiers. The best model was then selected according to the error rate and the parsimony of the model. A confusion matrix M = (m_ij_) was used to summarize the results from the best SVM model, where m_ij_ denotes the number of data points whose observed class is j and are assigned to class i by the classifier. In the confusion matrix, all correct classifications are located in the diagonal of the table and all misclassifications are represented by nonzero values outside the diagonal.

### Multinomial logistic regression classifier

In supervised learning, MLR is a classical multiclass classification method. Suppose we have *K* groups (*K* = activity categories in our study), which are represented by *Y* = 1, …, *Y = 0*. The MLR model has the form (Hastie et al. 2001; Menard 2009):


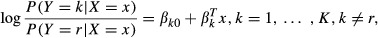
(1)

where *Y* = *r* is the reference group and *X* = *x* is the input vector. It is not difficult to show that the posterior probabilities conditional on the input are:


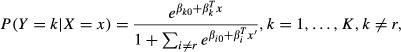
(2)

and


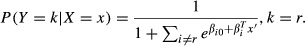
(3)

When we apply MLR to a classification problem, we assign the observation (minute-to-minute data in our study) to the group which has the largest posterior probability. In addition, from the formulas (2) and (3), we know that comparing the *K* posterior probabilities is the same as comparing the *K* − 1 linear combinations of 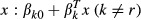, with 0. In other words, if we let


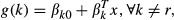
(4)

and


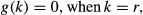
(5)

the group assignment can be done by


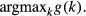
(6)

The MLR classifiers in this study were performed using SAS 9.2.

### Support vector machines classifier

The SVM classifier is an extension of the support vector classifier. It combines the features of the support vector classifier and the kernel method. The support vector classifier builds linear boundaries in the input feature space. However, the points in the input space cannot always be split by linear boundaries in the same space. In these situations, linear boundaries are sought in the high-dimensional feature space where all the points in the original input feature space are mapped into by a transformation. Using the kernel method, we gain access to the high-dimensional feature space through the inner product of the features in the original space, thus, bypass the computational burden of finding the image of the original input features in the high-dimensional space. The mathematical details of the support vector classifier, the kernel method, and SVM are provided in the Appendix.

### Use of SVM in a multiclass classification problem

The SVM is fundamentally a two-class classifier. However, the SVM can be extended to multiclass problems. Classifying multiple classes is commonly performed by combining multiple binary or two-class SVM classifiers. For a multiclass classification problem, either one-against-one voting scheme or one-against-all voting scheme can be used (Karatzoglou et al. 2006). In the one-against-one classification method, 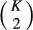 classifiers are built, where *K* is the number of classes. An observation will be tested on all of the classifiers and the observation will be assigned to the most frequently predicted class. In the one-against-all classification method, only *K* classifiers are constructed, and each of them separates one class from the rest of the *K* − 1 classes. An observation will be tested on all of the *K* classifiers and the observation will be assigned to the class whose corresponding classifier has the largest decision value. Although the one-against-one voting scheme is computationally intense, it has been shown to provide robust results with SVM classifiers. In this study, we implemented the one-against-one voting scheme.

### Parameter tuning in SVM

In order to find the best performance for the SVM classifier, the two parameters, so-called cost and *γ* (Dimitriadou et al. 2011), need to be tuned. The grid searching strategy (Karatzoglou et al. 2006) is used to search for the best combination of the parameters. The SVM classifier is tested on geometric sequences of combination of the values of the cost and *γ*, then the combination with the least 10-fold cross-validation error rate is selected as the best values for the cost and *γ*.

In the first step, the SVM classifier is tested on 44 combinations of the values of the cost and *γ*. The values for the cost come from the geometric sequence from 1 to 10^4^ by a factor of 10. The values for *γ* come from the geometric sequence from 10^−5^ to 10^5^ by a factor of 10.

From the above step, we obtain the best combination of the cost and *γ*. We fix this best value of the cost in the second step and tune *γ*. In this step, the values of *γ* come from the geometric sequence from γ* × 1.2^−5^ to γ* × 1.2^5^ by a factor of 1.2, where γ* is the best value of *γ* obtained from the first step. After the above two steps, we find the ultimate best combination of the cost and *γ*.

### Cross-validation

A classification model is assessed by its prediction error rate which is obtained by testing the model on independent testing samples. If the training sample is directly used to assess the performance of a classifier, we may obtain a result that is too optimistic (Hastie et al. 2001). In other words, the training error rate will be smaller than the prediction error rate. In order to obtain a legitimate estimation of the prediction error rate of a certain model, a multifold CV is often used. In a *n*-fold CV, the entire data set is separated into *n* sub-data sets with a roughly equal size. In a training-testing session, one of the *n* sub-data sets is reserved for testing, while the model is built on the rest of the *n*−1 sub-data sets. This kind of training-testing sessions is performed *n* times. Finally, the testing error rates of the training–testing sessions are combined to provide an estimate of the prediction error rate of the model. Generally speaking, a 5-fold or 10-fold CV will overestimate the true prediction error and thus is conservative and recommended by Hastie et al. (2001). In our application, a specific SAS macro was written to perform the 10-fold CV in MLR. The 10-fold CV of the SVM is performed using R package ‘e1071’ (Dimitriadou et al. 2011).

## Results

### Model development

**Figure 1:**
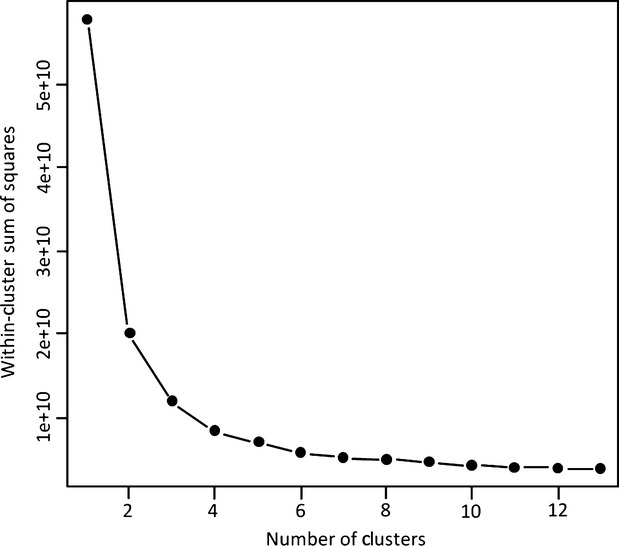
*K*-Mean clustering plot for accelerometer counts (act_X, act_Y, act_Z), steps, and position

Based on *K*-means clustering, the number of categories of the activities was determined using combinations of act_X, act_Y, act_Z, steps and position as the input features. The within-cluster sum of squares is provided in Figure 1. The figure shows an “elbow” around five clusters. Conventionally, it is recommended to retain the number of clusters up to the elbow, plus the first cluster following the elbow (Izenman 2008). Mathematically speaking, the number of the natural groupings of activities presented by the data is around six, which is less than the original eleven discrete activities designated in the protocol. Although the evidence provided by *K*-means clustering was purely mathematical, it clearly pointed out that a smaller number of activity groups is more appropriate for the current data structure. The data were therefore recategorized into six activity groups (Table 1).

**Table 1:** Physical activity categories

Activity category	Description	Position	Number of observations	Original categories
1	Sleep	Lying	2618	Sleep
2	Rest	Reclining	3035	Watching TV
3	Quiet play	Sitting	1747	Coloring, video game, puzzle
4	Low active play	Standing	1244	Kitchen/toys
5	Moderately active play	Standing	2569	Ball toss, active video game, dance, aerobics
6	Very active play	Standing	237	Running in place

Quasi-complete separation problems were detected in the data when applying the MLR model and we found the input feature position to be the reason. Therefore, the MLR classifiers were only implemented when the variable position is treated as a continuous variable and with position = 1 and position = 2 switched. In order to make the comparison with the MLR classifier, the input feature position was first treated as a categorical variable, then as a continuous variable in the SVM classifiers.

Altogether, we built 58 classification models (a detailed description of these models is available in the Appendix). Among these models, 52 models are based on from applying the MLR and SVM classifiers to the entire data set, and 6 models are based on applying the SVM classifier to the data without the sleep period.

When we applied the classifiers to the entire data set, the SVM classifiers performed better (have a smaller 10-fold CV error rate) than their logistic regression classifiers' counterparts. The overall 10-fold CV error rates of the MLR and SVM models applied to the data with and without the sleep period are presented in Table 2. Based on the entire data set, the SVM model PCO-18 with the input features act_X, act_Y, act_Z, vector magnitude, steps, and their 1-min and 2-min lag and lead values, and position (continuous) gave the least 10-fold CV error rate of 24.70%. The classification accuracy of this model is summarized in Table 3. Running in place (activity 6), which is always performed in a standing position and has large values of 3D-acceleration and steps, was nearly perfectly classified by the classifiers. On the other hand, rest was difficult to distinguish from sleep, since these activities can share the same accelerometer features. There were 2225 observations during sleep and 1004 observations during rest in which act_X = 0, act_Y = 0, act_Z = 0, steps = 0, and position = 0. Therefore, we decided to first remove the sleep periods, and then apply the SVM classifier since the accelerometer output during sleep (mainly zeros) is not informative. All the observations in the sleep period were categorized as activity 1 (sleep); therefore, all the removed observations were considered correctly classified. After applying this strategy, the error rates of the models and the overall classification error rates would be expected to decrease.

**Table 2:** The classification error rates of the models*

With Sleep Period				Without Sleep Period		
	
MLR		SVM		SVM		
			
Model	10-fold CV Error Rate (%)	Model	10-fold CV Error Rate (%)	Model	10-fold CV Error Rate (%)	Overall Classification Error Rate (%)
PCO-16	28.88	PCA-18	24.90	PCA-18	20.16	15.56
PCO-20	29.97	PCO-16	25.43	PCO-16	20.33	15.69
PCO-17	30.26	PCO-18	24.70	PCO-18	20.33	15.69
PCO-15	32.14	PCA-16	25.58	PCA-16	20.46	15.79
PCO-18	26.80	PCA-20	27.52	PCA-20	22.01	16.98
PCO-19	32.81	PCO-20	26.97	PCO-20	22.03	17.00

* A detailed explanation of the structure of the models used in this study can be found in the Appendix. The input feature position was treated either as a categorical variable (PCA) or a continuous variable (PCO). The input features of the models are given in the following:

Model Structure15: act_X + act_Y + act_Z + steps + lag/lead 1-min + position

Model Structure16: act_X + act_Y + act_Z + steps + lag/lead 1- and 2-min + position

Model Structure17: act_X + act_Y + act_Z + vm + steps + lag/lead 1-min + position

Model Structure18: act_X + act_Y + act_Z + vm + steps + lag/lead 1- and 2-min + position

Model Structure19: vm + steps + lag/lead 1-min + position

Model Structure20: vm + steps + lag/lead 1- and 2-min + position

**Table 3:** Classification accuracy

Activity	1	2	3	4	5	6
θ*(%)	91.44	65.66	74.07	68.49	93.73	98.73
π†(%)	31.55	17.30	33.03	24.92	1.91	0

*
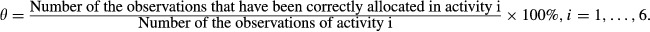

†
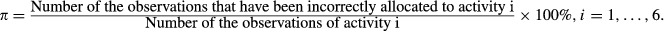

### Final model

The six best-performing SVM models were developed when we applied the classifier to the awake state only. The 10-fold CV error rates and the overall classification error rates of the SVM models ranged from 20.16% to 22.03% and from 15.56% to 17.00%, respectively. Compared to the best model applied to the entire data, the 10-fold CV error rates of these models were improved about 4% to 5%. When we applied the SVM classification model PCA-18 with the input features act_X, act_Y, act_Z, vector magnitude, steps, 1-min and 2-min lead and lag values, and position (categorical), we obtained the best overall classification error rate of 15.56% among all the models. The confusion matrix of this SVM Classifier is presented in Table 4.

**Table 4:** The confusion matrix

		Activity category					
		
		1	2	3	4	5	6
Predicted class	1	0*	0	0	0	0	0
	2	0	**2611**	300	20	3	0
	3	0	398	**1145**	397	52	0
	4	0	44	288	**768**	161	0
	5	0	2	14	59	**2349**	31
	6	0	0	0	0	4	**206**

* The (1,1)-entry of this matrix is zero, because activity category = 1 is sleep and we only applied the classifier to the data without sleep. There are actually 2618 observations in the sleep period, and those observations are considered to be correctly classified.

The bold values are the number of correctly-classified observations.

## Discussion

We have demonstrated that SVM can be used to correctly classify physical activities typical of preschool-aged children. To our knowledge, this is the first time that SVM has been applied to the classification of physical activities in preschool children. Using the SVM classifier, we achieved an overall classification error rate of 15.56% for the best model using a 10-fold CV. From the confusion matrix, we see that similar activities with close rankings are more difficult to classify than dissimilar activities.

SVM is an efficient and powerful supervised machine learning method. In SVM, we wish to predict the value of an outcome measure based on a number of input measures (Vapnik 1999). A supervised learning algorithm analyzes the training data and produces an inferred function, which is called a classifier. When the output variable is continuous, it yields a regression problem, whereas a categorical output variable yields a classification problem declaring group membership. The basic idea of a SVM classifier is to find an optimal maximal margin separating hyperplane or a decision boundary between two classes. Observations that fall on one side of the decision boundary are assigned to one class, and observations that fall on the other side are assigned to the other class. Such a decision function can be expressed by a mathematical function of an input vector *x* = (*x*_1_, …, *x*_*p*_), the value of which is the predicted label of *x* (either +1or −1 for a two-class problem). The classifier can therefore be written as *g*(*x*) = *sign*(*f*(*x*)), where *f*(*x*) = *b* + *w*^*T*^*φ*(*x*) and 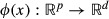 is a transformation from the original input feature space to a high-dimensional space where the points are linear separable. In this way, we have parameterized the function by the weight vector *w* and the scalar *b*. More generally, the SVM classifier can be stated as finding a solution to an optimization problem (Burges 1998; Hastie et al. 2001; Smola and Schölkopf 2004; Steinwart and Christmann 2008). The goal is to locate a decision boundary, using information from the predictors, so that the partitions are as homogenous as possible. Unlike classification and regression trees (CART), SVM does not classify observations in the input space in a stage-wise fashion, and only observations near the classification boundary that are difficult to classify determine the criterion by which classes are to be assigned. ANN modeling has been applied to physical activity classification in school-aged children (De Vries et al. 2011a) and similar classification accuracy to our results was achieved. However, compared to ANN, SVM can provide a clear boundary between the two classes in the input feature space and this boundary can be used in future investigations. SVM is fundamentally a two-class classifier, but it can be extended to multiple class problems. Classifying multiple classes is commonly performed by combining multiple binary or two-class SVM classifiers and the final classifier is the one that dominates the most.

Compared with SVM, MLR is a commonly used classifier. It has been successfully implemented in various situations (Hossain et al. 2002; Wang 2005; Torres et al. 2009). However, in our study, application of the MLR classifiers to physical activities of preschoolers was not without difficulties. The quasi-complete separation problem (Albert and Anderson 1984; Santner and Duffy 1986; Allison 2008) prevented us from applying MLR classifiers to the data when position was treated as a categorical input feature. The problem hindered our application of MLR simply because if a quasi-complete separation problem is present, the maximum likelihood estimate does not exist (Albert and Anderson 1984). Thus, no optimum boundary can be established between categories. Even when treating position as a continuous variable, we had to recode it to avoid such a problem. Although the MLR model is easy to interpret and available in commonly used statistical software, the quasi-complete separation problem we met hindered its application in the data we used.

The SVM classifiers, on the other hand, have been shown to be powerful in our application. However, to obtain the best values for the parameters in each of the SVM classifiers, the model had to be tuned. The parameter tuning process was computationally intense. It can take days to tune the parameters of a SVM classifier on a regular laptop or desktop. Compared with SVM classifiers, the MLR classifier was computationally simpler. Although the parameter tuning process was time consuming, once the optimal values for the parameters are obtained, no further tuning is needed when applying the model to classify new observations. Based on the 10-fold cross-validation error rates of the SVM classifiers we obtained, there were only minor differences between treating the input feature position as a continuous variable or a categorical variable in the classification models. However, this does not imply that there is a linear relationship between the values of position and the activity type. Instead, this is a feature brought about by the SVM classifier which is insensitive to the type of the input features, whether they are categorical or continuous.

The minute-to-minute accelerometer counts, even with additional information of steps and position, could not accurately distinguish rest from sleep. In practice, oftentimes sleep is identified visually from the pattern of consecutive zero accelerometer counts during the night-time. Also, participant activity records are used to identify night-time sleep and day-time naps. In our application, we evaluated the MLR and SVM models with and without sleep, verified by continuous observation by our staff. The error rates of the SVM classifiers would be expected to improve once the data were partitioned into sleep and awake states.

Although our best SVM model utilizes all of the input features (act_X, act_Y, act_Z, vector magnitude, steps, their 1- and 2-min lead/lag values, and position), the more parsimonious model, which incorporates only the vector magnitude, steps, their 1- and 2-min lead/lag values, and position performed relatively well (Model S-PCA-20 and S-PCO-20). This is not beyond our expectation. The vector magnitude and the 3D accelerations are highly correlated: the Pearson correlations between the vector magnitude and the accelerations on the X, Y, and Z axis are 0.96, 0.94, and 0.95, respectively. In addition, the final categories of activities we used in this study differ on the degree of acceleration rather than on the direction of the movement. In addition, the 1-min and 2-min lag and lead values contribute to our models. Naturally, a series of movements is more informative than a point value to classify activities. It is difficult to tell accurately what kind of activity a person is performing from only the snapshot of the 3D acceleration readings. Since activities are frequently correlated from one moment to the next, the lag and lead values capture this aspect of activity duration. For example, both moderately active activities (like dancing) and very active activities (like running) can have overlapping acceleration readings, but running has a longer duration of high accelerations, while dancing has a shorter duration of high accelerations.

SVM models have been used in physical activity classification in adults (Cho et al. 2008; He and Jin 2008). High classification accuracy (92% on average) was achieved by He and Jin (2008) using autoregressive-based features extracted by fitting an autoregressive model to the acceleration activity signals, but the activities (running, resting, jumping, and walking) differed substantially which facilitates classification. Considering the similarity between several activity types in our study, the classification accuracy of the SVM models is quite good.

To apply the established SVM classification model to classify new ActiGraph GT3X+ observations, there is no need to explicitly program all the separating hyperplanes. We encourage researchers to tune the SVM classifier in R using the *tune.svm* function in the package ‘e1071’ (Dimitriadou et al. 2011), then to classify new observations in R using the *predict* function. For future applications, the user can save the SVM model (object) produced by the package ‘e1071’ using the *save* function. Then, for new observations, the user can apply the *load* function to reload the previously established model and classify new observations, rather than reloading the training data and tuning the parameters again. In this way, researchers can share their models without providing the original training data. The R objects of our best models with and without the sleep period in this study can be obtained per request.

In conclusion, SVM supersedes the classical classifier MLR in categorizing physical activities in preschool-aged children. Using accelerometer data, SVM can be used to correctly classify physical activities typical of preschool-aged children with an acceptable classification error rate.

## Figures and Tables

**Figure A1:**
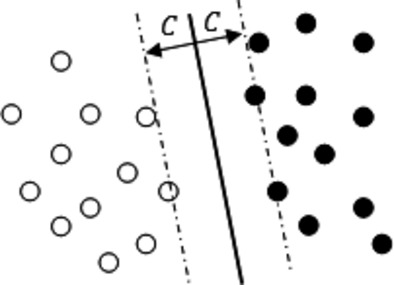
The separating hyperplane and the margin. The different colors show points from different classes. The solid line shows a hyperplane that separates the two classes. The dotted lines indicate the edges of the two classes. The double-sided arrows show the margin (2*C*).

**Figure A2:**
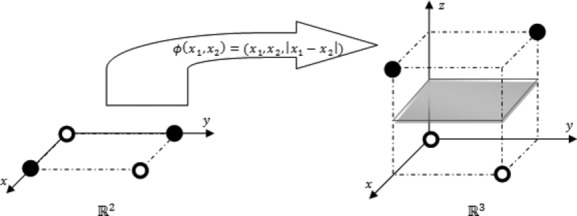
A transformation example. The points in white are from class 1, and the points in black are from class 2. The parallelogram in gray shows a plane that separates the two classes.

## Appendix: The Mathematical Details of the Support Vector Classifier, the Kernel Method, and SVM

The SVM classifier is an extension of the support vector classifier. It combines the features of the support vector classifier and the kernel method.

### Support vector classifier

Consider a two-class training set of *N* observations (*x*_1_, *y*_1_), (*x*_2_, *y*_2_), …, (*x*_*N*_, *y*_*N*_), where  are the input features, and *y*_*i*_ = {−1, 1}, *i* = 1, …, *N* are the class assignments. If the two classes of points are linear separable, then at least a hyperplane, which is defined by

(A1)

can be found. Then, the classification can be done by

(A2)

Since the two classes are linear separable, the best hyperplane that separates the two classes has the largest margin between the points of class *y* = 1 and *y* = −1 (Fig. A1). Actually, the task of finding the best hyperplane can be expressed as the following optimization problem (Hastie et al. 2001):

(A3)

subject to .

The two times of *C* in equation (A3) is the margin, as shown in Figure A1.

If the two classes cannot be perfectly separated, then the constraint in equation (A3) can be modified by allowing some points to stay at the wrong side of the margin. Then, consider the overlapping cases and drop the norm constraint on *β*, the optimization problem can be rewritten as follows:

(A4)

subject to , and ∑_*i*_*ζ*_*i*_ ≤ constant.

Solving this optimization problem, we obtain the estimates for *β*_0_ and *β*:  and  (Burges 1998; Hastie et al. 2001; Smola and Schölkopf 2004). Since the estimate of *β* is only supported by the points on the edge of the margin and on the wrong side of the margin, those points are called support vectors (Hastie et al. 2001; Steinwart and Christmann 2008). Finally, given  and , the classification can be achieved by

(A5)

### Kernel method

The support vector classifier builds linear boundaries in the input feature space. However, the points in the input space cannot always be split by linear boundaries in the same space. In these situations, linear boundaries are sought in the high-dimensional feature space where all the points in the original input feature space are mapped into by a transformation.

Consider, for example, four points in : (0,0), (0,1), (1,0), and (1,1). Among them, (0,0) and (1,1) are from class 1; (0,1) and (1,0) are from class 2. One may find the curves that can separate the two classes. But in , linear boundaries are lines. We are not able to find a linear boundary for the two classes in . Now, consider the transformation , such that

(A6)

Under the transformation, (0,0), (0,1), (1,0), and (1,1) are mapped to (0,0,0), (0,1,1), (1,0,1), and (1,1,0). Now, the two classes can be separated in  – any plane that is parallel to the *xy* plane with a *z*-intercept between 0 and 1 can perfectly separate the two classes (Fig. A2).

In some cases, the formula we want to evaluate involves the transformation  only through the form of its inner product. In such a situation, if we define a function , such that

(A7)

for all , then we do not have to first transform all the points. Instead, we can work in the original sample space through the newly defined kernel function *K*.

For SVM, instead of using the original data points *x* as the input features, the SVM classifier uses basis expansion of the original ones: ϕ (*x*_*i*_) = (ϕ_1_(*x*_*i*_), …, ϕ _*d*_(*x*_*i*_)), *i* = 1, …, *N*, as the input features. With sufficient basis functions, the data would finally be linear separable in the enlarged input space.

In SVM, the optimization problem of finding the best separation hyperplane involves the transformation ϕ(*x*) only through its inner product (*x*_*i*_)^*T*^ ϕ(*x*_*i*_). Indeed, if we let , we do not have to worry about the computation burden from *x* to ϕ(*x*) – finding the coordinates in the high-dimensional space. We can deal with the kernel function ) directly in the original input space.

Several kernel functions are available. The popular choices are (Hastie et al. 2001; Steinwart and Christmann 2008; Dimitriadou et al. 2011):

(A8)

(A9)

(A10)

In this study, we used the radial basis kernel.

### The Model Description

We established 58 models in total. The model structures are shown in Table A1. The 10-fold CV error rates are provided in Table A2. In Table A2, the six models shown in bold characters are those ones which were built on the data without sleep period. In Table A2, S stands for SVM and L indicates MLR. The input feature position was treated either as a continuous variable (PCO) or a categorical variable (PCA). The number after the second dash (or the first one, if there is no indicator for position) tells us which model structure was used.

**Table A1:** The model structures

Model structure	Description
Model Structure 1	activity.category ∼ act_X + act_Y + act_Z
Model Structure 2	activity.category ∼ act_X + act_Y + act_Z + vm
Model Structure 3	activity.category ∼ act_X + act_Y + act_Z + steps
Model Structure 4	activity.category ∼ act_X + act_Y + act_Z + position
Model Structure 5	activity.category ∼ act_X + act_Y + act_Z + vm + steps
Model Structure 6	activity.category ∼ act_X + act_Y + act_Z + vm + position
Model Structure 7	activity.category ∼ act_X + act_Y + act_Z + steps + position
Model Structure 8	activity.category ∼ act_X + act_Y + act_Z + vm + steps + position
Model Structure 9	activity.category ∼ vm + steps
Model Structure 10	activity.category ∼ vm
Model Structure 11	activity.category ∼ steps
Model Structure 12	activity.category ∼ vm + steps + position
Model Structure 13	activity.category ∼ vm + position
Model Structure 14	activity.category ∼ steps + position
Model Structure 15	activity.category ∼ act_X + act_Y + act_Z + steps + position + lag/lead 1-[act_X + act_Y + act_Z + steps]
Model Structure 16	activity.category ∼ act_X + act_Y + act_Z + steps + position + lag/lead 1/2-[act_X + act_Y + act_Z + steps]
Model Structure 17	activity.category ∼ act_X + act_Y + act_Z + vm + steps + position + lag/lead 1-[act_X + act_Y + act_Z + vm + steps]
Model Structure 18	activity.category ∼ act_X + act_Y + act_Z + vm + steps + position + lag/lead 1/2-[act_X + act_Y + act_Z + vm + steps]
Model Structure 19	activity.category ∼ vm + steps + position + lag/lead 1-[vm + steps]
Model Structure 20	activity.category ∼ vm + steps + position + lag/lead 1/2-[vm + steps]

The model structures were developed in a step-wise manner: first, we included the triaxial accelerometer outputs (act_X, act_Y, act_Z) from the device in the model structure (model structure 1). Then, we gradually included other features (vm, steps, and position) in the subsequent model structures (model structure 2–8). Since vm is a summary of the triaxial accelerometer outputs, we built a model structure based on only vm (model structure 10). Steps are another important feature and thus we built a model structure (model structure 11) based on it. Next, we added more features (steps and position) to vm and/or steps and developed model structures 9, 12–14. Finally, we added the lag and lead values of the input features to the best-performing models (model structures 15–20).

**Table A2:** The performance of the model

Rank	Model	10-fold CV error rate (%)	Rank	Model	10-fold CV error rate (%)
1	**S-PCA-18**	**20.16**	30	S-PCA-12	34.57
2	**S-PCO-18**	**20.33**	31	S-PCO-6	35.91
2	**S-PCO-16**	**20.33**	32	S-PCA-6	36.06
4	**S-PCA-16**	**20.46**	33	L-PCO-8	36.31
5	**S-PCA-20**	**22.01**	34	S-PCO-4	36.56
6	**S-PCO-20**	**22.03**	35	S-PCA-13	36.56
7	S-PCO-18	24.70	36	S-PCA-4	36.600
8	S-PCA-18	24.90	37	S-PCO-13	36.78
9	S-PCO-16	25.43	38	L-PCO-7	36.98
10	S-PCA-16	25.58	39	S-PCA-14	37.14
11	L-PCO-18	26.80	40	S-PCO-14	37.21
12	S-PCO-20	26.97	41	L-PCO-12	37.43
13	S-PCA-20	27.52	42	S-5	39.11
14	S-PCO-17	28.09	43	S-3	39.49
15	S-PCA-17	28.73	44	L-PCO-6	39.50
16	L-PCO-16	28.88	45	L-5	39.89
17	S-PCO-15	28.95	46	L-PCO-4	39.90
18	S-PCA-15	29.30	47	S-9	39.99
19	S-PCA-19	29.42	48	L-PCO-13	41.12
20	S-PCO-19	29.42	49	S-2	41.32
21	L-PCO-20	29.97	50	L-3	41.78
22	L-PCO-17	30.26	51	S-1	41.73
23	L-PCO-15	32.14	52	L-9	42.01
24	L-PCO-19	32.81	53	L-PCO-14	42.01
25	S-PCA-8	33.73	54	L-2	43.00
26	S-PCO-8	33.84	55	L-1	45.00
27	S-PCO-7	34.15	56	L-10	45.71
28	S-PCA-7	34.18	57	S-11	46.2
29	S-PCO-12	34.55	58	L-11	46.47

The bold values are the number of correctly-classified observations.
